# Single dose testosterone increases total cholesterol levels and induces the expression of HMG CoA Reductase

**DOI:** 10.1186/1747-597X-7-12

**Published:** 2012-03-20

**Authors:** Nina Gårevik, Cristine Skogastierna, Anders Rane, Lena Ekström

**Affiliations:** 1 Department of Laboratory Medicine, Division of Clinical Pharmacology, Karolinska Institutet, Karolinska University Hospital, SE-14186 Stockholm, Sweden

**Keywords:** Testosterone, Cholesterol, HMG CoA reductase

## Abstract

**Background:**

Cholesterol is mainly synthesised in liver and the rate-limiting step is the reduction of 3-hydroxy-3methylglutaryl coenzyme A (HMG-CoA) to mevalonate, a reaction catalysed by HMG-CoA reductase (HMGCR). There is a comprehensive body of evidence documenting that anabolic-androgenic steroids are associated with deleterious alterations of lipid profile. In this study we investigated whether a single dose of testosterone enanthate affects the cholesterol biosynthesis and the expression of HMGCR.

**Methods:**

39 healthy male volunteers were given 500 mg testosterone enanthate as single intramuscular dose of Testoviron^®^--Depot. The total cholesterol levels prior to and two days after testosterone administration were analysed. Protein expression of HMGCR in whole blood was investigated by Western blotting. In order to study whether testosterone regulates the mRNA expression of HMGCR, *in vitro *studies were performed in a human liver cell-line (HepG2).

**Results:**

The total cholesterol level was significantly increased 15% two days after the testosterone injection (p = 0.007). This is the first time a perturbation in the lipoprotein profile is observed after only a single dose of testosterone. Moreover, the HMGCR mRNA and protein expression was induced by testosterone *in vitro *and *in vivo*, respectively.

**Conclusion:**

Here we provide a molecular explanation how anabolic androgenic steroids may impact on the cholesterol homeostasis, i.e. via an increase of the HMGCR expression. Increasing knowledge and understanding of AAS induced side-effects is important in order to find measures for treatment and care of these abusers.

## Background

Anabolic androgenic steroids (AAS) including testosterone, other endogenous androgenic hormones and synthetic substances structurally related to these compounds are the most frequently detected doping agents in the society and sports. The abuse of these agents for cosmetic purposes among non-competitive recreational body-builders and non-athletes is a considerable health concern. According to studies in Western societies the prevalence of abuse of anabolic androgenic steroids among high school and college students ranges from 1 to 5% [1-3].

There is a comprehensive body of evidence documenting that AAS induce various deleterious alterations of the lipoprotein profile. The most prominent changes include elevations of low density lipoprotein (LDL) and decreases of high density lipoprotein (HDL) [4-7]. The long-term consequences of these alterations are still unknown but it is possible that the perturbation of the lipid profile may be associated with an increase in risk of coronary artery disease.

Cholesterol is mainly synthesised in the liver and the rate-limiting step is the reduction of 3-hydroxy-3methylglutaryl coenzyme A (HMG-CoA) to mevalonate, a reaction catalysed by HMG-CoA reductase (HMGCR). Normally in mammalian cells the transcription of *HMGCR *is suppressed by cholesterol derived from the internalization and degradation of LDL via the LDL receptor. Competitive inhibitors of the HMGCR by statins lead to induction of the expression of LDL receptors in the liver, which in turn increases the catabolism of plasma LDL and lowers the concentration of cholesterol in plasma. It is conceived that statins have a preventive effect on cardiovascular disease to a great extent by these mechanisms in several populations [8].

In this study we investigated whether a single dose of testosterone enanthate affects the cholesterol profile and the expression of HMGCR in healthy volunteers. The lipoprotein profile was analysed prior to, and two and fifteen days after administration of 500 mg testosterone enanthate. The protein expression of HMGCR in whole blood was determined by Western blotting. Moreover, human liver cells (HepG2) were exposed to supra-physiological concentrations of testosterone enathate and the mRNA HMGCR level was quantified by real time analysis.

## Methods

### Subjects and design

Study subjects included 39 healthy volunteers originating from the study population described in detail elsewhere [9]. All participants were males at age 18-50 years, and gave informed consent consistent with the approval of the Ethics Review Board. The participants were given 500 mg testosterone enanthate as a single intramuscular dose of Testoviron^®^-- Depot (kindly provided by Schering Nordiska AB, Solna) equivalent to 360 mg testosterone. Blood and serum was collected prior to (day 0), 2 and 15 days after testosterone administration. All samples were collected between 07 and 11 am and were directly frozen at -20°c.Averse drug reactions (ADRs) were monitored from the time of screening until day 15 after administration of testosterone. The study was conducted according to the Helsinki declaration and the ICH Harmonised Tripartite Guideline for Good Clinical Practice.

### Western blotting

The level of the HMGCR enzyme in whole blood samples was examined by Western blotring analysis. Frozen whole blood samples were available from 24 subjects of the 39 individuals included in the study. The blood samples were mixed 1:12 with 2 mM EDTA and complete protease inhibitor cocktail (cat no 11 697 498 001 Roche) and freeze-thawed three times. The hemolysates were separated on 12% polyacrylamide gel, electrotransferred onto Hybond-C extra membrane (GE healthcare), blocked overnight in high salt base buffer (HSB) (50 mM Tris-HCl/500 mM NaCl, pH 7.5), 2% dried milk, 1% BSA and incubated for two hours with 1:400 dilution of rabbit anti-HMGCR antibody (Santa Cruz Biotechnology). Membranes were washed in HSBT (HSB with 0.05% Tween) and incubated for 1 hour with 1:2500 horseradish peroxidise-linked mouse anti-rabbit immunoglobulin antibody (Promega, WI, USA). Detection of the protein bands was carried out using ECL Plus Western Blotting Detection System kit (GE healthcare, Uppsala, Sweden) and quantified by densitometry using BioRad Quantity One1 (version 4.1 software). A standard curve with increasing concentration of liver microsomes was included on each gel. The HMGCR expression in whole blood was normalized against the total protein level as was determined by Lowry [10].

### Cell culture

HepG2 cells were cultured in MEM supplemented with 5% FCS, 1% penicillin/streptomycin, 1% L-glutamine and maintained in humidified atmosphere at 37°C and 5% CO_2_. Prior to testosterone treatment the HepG2 cells were split and plated in 6-well plates and pre-incubated for 3 days. Testosterone enanthate was diluted in ethanol and added to the cells (1 μM) for 2-48 hours. The non-treated controls were incubated with vehicle only. The experiments were performed in four independent experiments. For RNA extraction the cells were harvested with Trizol (Invitrogen, UK) and kept at −80°C until analysis.

### RNA extraction and cDNA

Total RNA extraction was performed using 0.5 ml Trizol per well according to manufacturer´s instructions. RNA (0.5 μg) was reverse transcribed into cDNA with hexamer primer using first-strand cDNA synthesis kit (Amersham Biosciences, NJ, USA) according to the manufacturer´s protocol and diluted 10 X in sterile water.

### Real time PCR

The mRNA level of HMGCR in testosterone treated HepG2 cells was determined by real-time PCR. Beta-actin (PN 4326315E, Applied Biosystems, Foster City, CA) was chosen as an endogenous housekeeping control gene. Quantitative real-time PCR was performed using the 7500 Fast system, Applied Biosystems. Reaction mixtures contained Taqman reaction mix (Applied Biosystems), HMGCR Taqman Assay mix (Hs01103000_m1, Applied Biosystems), 4 μl cDNA template in a total volume of 20 μl. Thermal cycling conditions included activation at 95°C (10 min) followed by 40 cycles each of denaturation at 95°C (15 sec) and annealing/elongation at 60°C (1 min). Each reaction was performed in triplicates and no-template controls were included in each experiment. The untreated sample was employed as a calibrator and the ΔΔ CT-formula was used as described previously [11]. The gene expression was quantified as the yield of the target gene relative to that of Beta-actin gene.

### Serum analysis

Serum total cholesterol, HDL, LDLD, alanine transaminase (ALAT) and aspartate transaminase (ASAT) were determined by routine analyses at the division of clinical chemistry, Karolinska University Hospital. Total testosterone levels in serum prior to and after testosterone administration had been measured by GCMS in a previous study [12]

### Data analysis

Statistical analyses were performed using GraphPad Prism software 4.03 (GraphPad, San Diego, CA). The increase in cholesterol levels after testosterone injection was compared using ANOVA. The protein expression of HMGCR prior to and after testosterone administration was compared using Wilcoxon test whereas Mann-Whitney test was used for comparison of HMGCR mRNA levels between testosterone treated and non-treated HepG2 cells.

## Results

### Cholesterol levels

**Figure 1:**
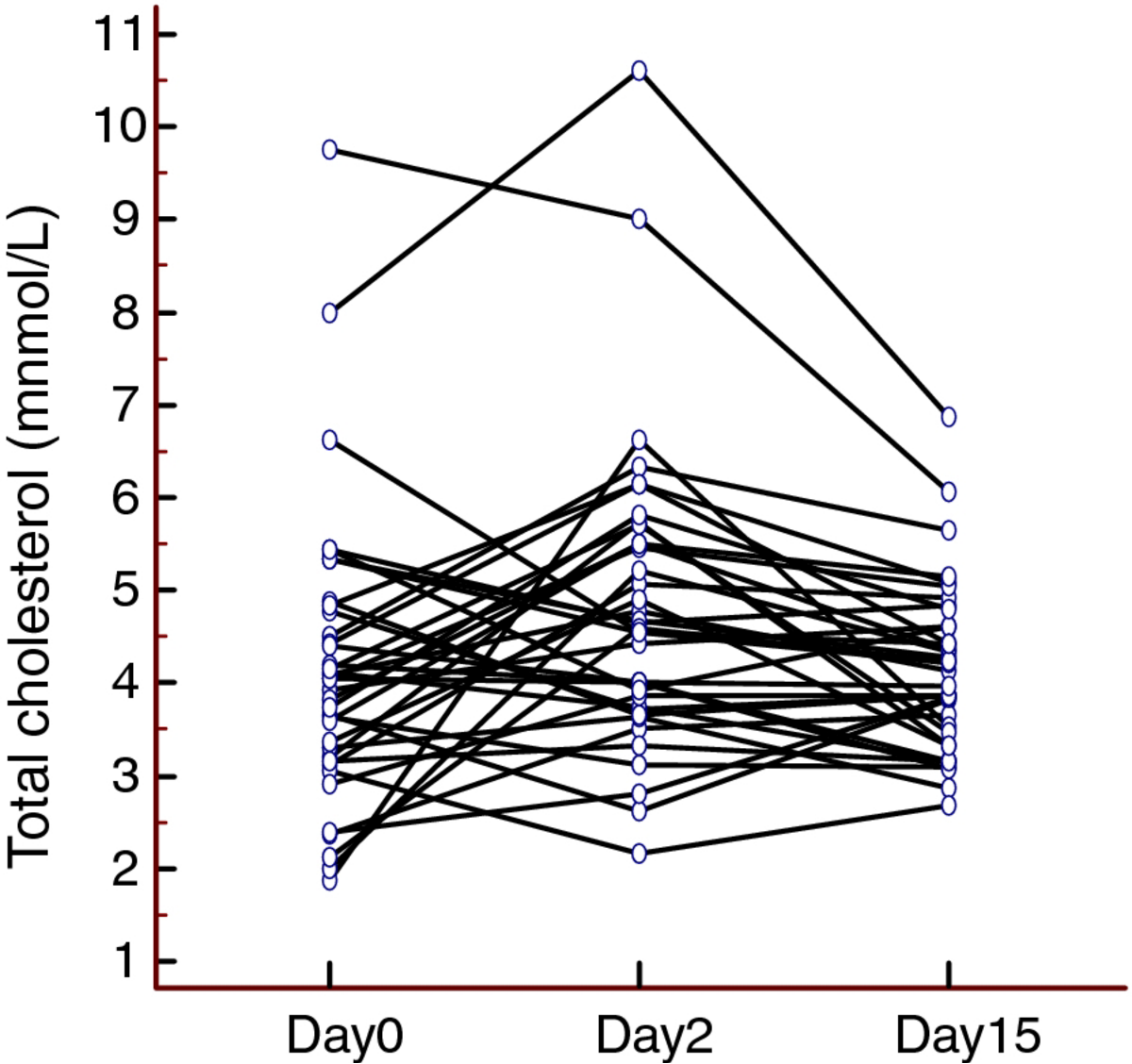
**The change in total cholesterol levels on day 0 (prior to testosterone administration), 2 and 15 days after testosterone administration**. The mean total cholesterol level was significantly higher 2 days after testosterone administration as compared to on day 0 (p = 0.007; ANOVA). 15 days after testosterone administration the total cholesterol level was back to baseline.

Total cholesterol level increased on day two (mean 4.87 mmol/L +/SD 0.25) compared to day 0 (mean 4.23 mmol/L +/− SD 0.27) (p = 0.007; ANOVA) (Figure 1). On day 15 the total cholesterol level was back to baseline (mean 4.23 +/− SD 0.14). There was no significant difference in HDL, LDLD or VDL between day 0 and day 2 (data not shown).

A correlation analysis demonstrates that the increase in cholesterol levels was significantly correlated to the total testosterone levels on day 2 (r^2 ^= 0.14 p = 0.02; Spearman´s rank correlation test). There were no alterations in the liver function biomarkers ALAT and ASAT.

### HMGCR expression in whole blood

**Figure 2:**
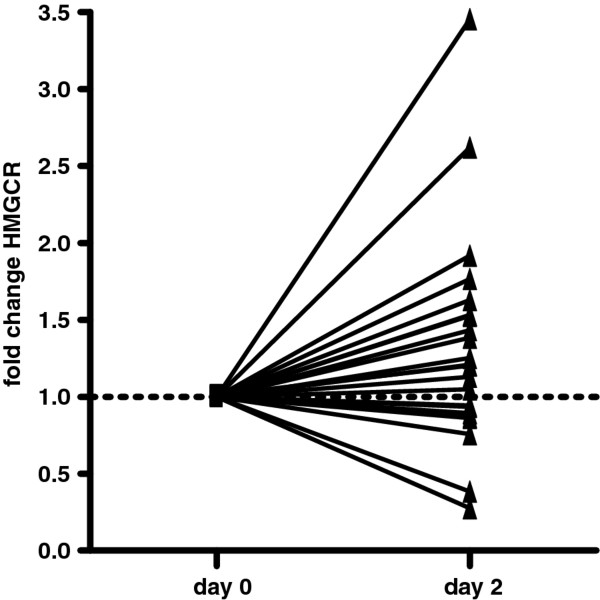
**HMGCR protein levels *in vivo***. The fold change in HMGCR protein expression in whole blood on day 2 was assessed by Western blotting. The individual expression values on day 2 were presented in relation to the level on day 0 which was set to one. There was a significant increase in HMGCR level on day 2 (p = 0.03, Mann Whitney test).

The increase in cholesterol level prompted us to investigate the expression of the main enzyme involved in the cholesterol synthesis. The protein expression of HMGCR, the rate limiting enzyme in the cholesterol synthesis, was investigated by Western blotting in whole blood from the subjects prior to, and two days after testosterone administration. In 80% of the individuals the HMGCR expression was increased 2 days after the administration of testosterone (Figure 2). In the whole group, there was a significant increase in HMGCR expression on day two compared to day 0 (p = 0.03; Mann Whitney test).

### HMGCR mRNA expression in HepG2 cells

**Figure 3:**
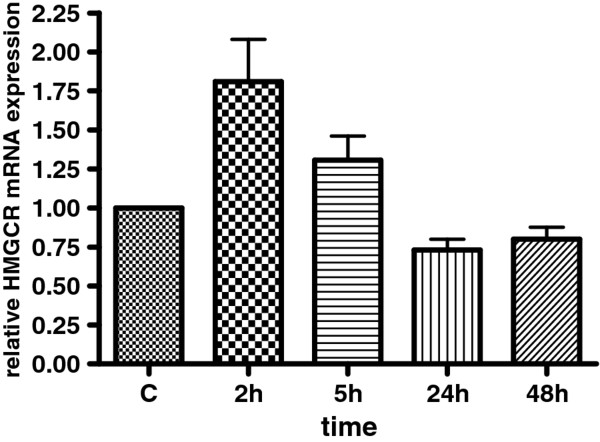
**HMGCR mRNA levels *in vitro***. The effect of testosterone enanthate on HMGCR mRNA levels in HepG2 cells was determined at different time points. Cells were incubated with testosterone enanthate or vehicle (C = control set as 1) and total RNA was extracted, reverse transcribed and subjected to real-time PCR. Measurements were performed in triplicates and data are presented as means ± SD (N = 4). There was a significant increase in HMGCR mRNA expression 2 hours after testosterone exposure (p < 0.001; Mann Whitney test), whereas after 24 hours the levels was significant lower (p = 0.047) as compared to the controls.

Since testosterone induces HMGCR expression *in vivo*, we investigated whether it would also affect the mRNA expression of HMGCR *in vitro*. HepG2 cells were exposed to testosterone enanthate (1 uM) for 2-48 hours. The gene expression of HMGCR was 1.8 fold higher after 2 hours testosterone treatment (p < 0.001; Mann Whitney test (Figure 3). A significant 0.8 fold decrease in HMGCR mRNA was observed after 24 hours (p = 0.047). After 48 hours the mRNA levels were back to basal level.

## Discussion

Even though accumulating evidence indicates that testosterone may have adverse effect on the lipoprotein profile and cardiovascular health, this is, to the best of our knowledge, the first time an increase in total cholesterol level has been observed after only one single dose of testosterone. 15 days after the testosterone administration the cholesterol levels in the volunteers was back to baseline levels.

Numerous reports on the effects of AAS on lipoproteins in humans have been published in the last 25 years. To a large extent the results of these studies present consistent results, i.e. AAS cause a marked depression in serum HDL and an elevation in LDL (as reviewed by [13] and [14]). However, for the effect on total cholesterol levels the results are conflicting. Some studies have found that repeated supra-physiologic doses of AAS is associated with an increase in total cholesterol levels [15,16], whereas others have failed to find such an association [5,6]. The reason for the discrepancy observed in the effect on total cholesterol after AAS administration may be the different study designs used, sampling time, type of AAS used, administration route etc. However, most importantly, these studies all concerned subjects with multiple doses or chronic use of AAS.

The baseline serum testosterone concentration in our study population was 5 ng/ml [12] which is the same as has been seen in other study groups similar in respect of age, gender, ethnicity [17]. Two days after testosterone administration the serum testosterone level has increased by 200% [12]. Here we show that circulatory concentration of total testosterone was significantly associated with the increase in cholesterol levels on day 2. No toxic effect on liver function, as assessed by ALAT and ASAT, was found indicating that the increase in total cholesterol observed is directly associated with the administration of testosterone and not an artificial effect of the injection, i.e. stress or impact on the liver function.

The synthesis of cholesterol is dependent on the activity of HMGCR, and we therefore investigated if testosterone could affect the expression of this enzyme. Here we show for the first time that a supra-physiological dose of testosterone induces the expression of HMGCR *in vivo *in healthy volunteers. These observations were supported by the results from the experiments with HepG2 cells exposed to 1 μM testosterone. This concentration is in the range of the levels achieved after administration of testosterone to the volunteers [12], and yielded a marked induction of the transcription of the HMGCR gene. Further support for this testosterone effect is provided from experiments in castrated mice and male Sprague-Dawley rats in which 14 days administration of AAS (testosterone and nandrolone) led to an up-regulation of meibomian and adrenal gene expression of HMGCR, respectively [18,19]. The mechanisms for the androgen induced up-regulation of HMGCR transcription as well as the physiological consequences have not been investigated and needs to be further elucidated. High cholesterol levels are known to exert a negative feedback on the cholesterol synthesis on a transcriptional level [20,21]. This may explain the time dependent response observed in our HepG2 experiments, i.e. the HMGCR mRNA expression was normalized or even down-regulated 24 hours after testosterone treatment.

Other molecular mechanisms behind the unfavourable effects of AAS on the lipoprotein profile have not been well investigated. It is believed that AAS exert some of their influence on the cholesterol profile by inducing the HDL-catabolising enzyme hepatic triglyceride lipase (HTGL) synthesis in the liver [5]. A 143-232% increase of HTGL activity has been described during AAS abuse [4]. Here we provide an additional molecular explanation how AAS may impact on the cholesterol homeostasis, i.e. via an increase of the HMGCR expression. Further studies may provide yet other explanations behind the lipid profile perturbation observed as a result of AAS abuse.

AAS are the most commonly used doping agents in athletes (http://www.wada-ama.org). However, the abuse of AAS is not limited to competitive sports, but has spread to sport amateurs and non-athletes and is considered a serious concern in the society [2,22]. It is therefore urgent to study and establish the awareness of the adverse effects caused by AAS. Here we provide scientific evidence that a supra-physiological dose of testosterone may have adverse effects on the cholesterol metabolism. Our setting is, however, very different from the situation for the illicit users that typically take AAS in repeated courses known as "cycles", each lasting several weeks to several months. One might suspect that the HMGCR expression will rapidly increase after each dose, keeping the HMGCR protein expression and cholesterol metabolism elevated continuously during the "cycles".

Some limitations of our study need to be addressed. Using whole blood as surrogate model for HMGCR expression may not reflect the expression profile in the liver. Nevertheless, blood is a natural surrogate organ in the absence of availability of liver biopsies. It is to be noted that HMGCR and its main important transcription factors, i.e. SREBP-2 are highly abundant in the blood cells further supporting that whole blood may be used for expression analysis. Another limitation is that we only have the cholesterol profile in our study participants. The inclusion of cholesterol activity biomarkers such as lathosterol would further support that the administration of supra-physiological doses of testosterone disturb the cholesterol metabolism *in vivo*. Unfortunately our study design did not permit further analysis of additional biomarkers.

## Conclusions

In conclusion, we have shown that already *one single dose *of testosterone enanthate increases the serum total cholesterol level. This *immediate *response to AAS is a cause for concern and signals that the lipid metabolic perturbation is a rapid response and warrants close follow-up of the cardiovascular risk factors that may appear later in life in abusers. There are many cases described in the literature on such adverse events in *heavy *abusers of AAS. Our findings demonstrate that such effects may occur also in subjects with moderate, intermittent, or temporary abuse of AAS. Therefore, given our findings, public efforts should be centered on primary prevention.

## Competing interests

The authors declare that they have no competing interests.

## Authors' contributions

LE and AR planned, designed and wrote the study protocol for the *in vivo *study. CS planned and performed the cell culture analysis. LE and NG interpreted the data. NG performed the statistical analysis and managed the sample collection. LE wrote the first draft of the manuscript. All authors contributed to and have approved the final manuscript.
